# Seroepidemiological Study of Interepidemic Rift Valley Fever Virus Infection among Persons with Intense Ruminant Exposure in Madagascar and Kenya

**DOI:** 10.4269/ajtmh.15-0383

**Published:** 2015-12-09

**Authors:** Gregory C. Gray, Benjamin D. Anderson, A. Desirée LaBeaud, Jean-Michel Heraud, Eric M. Fèvre, Soa Fy Andriamandimby, Elizabeth A. J. Cook, Saidi Dahir, William A. de Glanville, Gary L. Heil, Salah U. Khan, Samuel Muiruri, Marie-Marie Olive, Lian F. Thomas, Hunter R. Merrill, Mary L. M. Merrill, Juergen A. Richt

**Affiliations:** Division of Infectious Diseases, Duke Global Health Institute, Duke University, Durham, North Carolina; Nicholas School of the Environment, Duke University, Durham, North Carolina; Emerging Pathogens Institute, University of Florida, Gainesville, Florida; Division of Pediatric Infectious Diseases, Stanford University, Palo Alto, California; Virology Unit, Institut Pasteur de Madagascar, Antananarivo, Madagascar; Institute of Infection and Global Health, University of Liverpool, United Kingdom; International Livestock Research Institute, Nairobi, Kenya; Centre for Microbiology Research, Kenya Medical Research Institute, Kenya; Division of Vector Borne and Neglected Tropical Diseases, Ministry of Health, Nairobi, Kenya; Department of Environmental Sciences, Technical University of Mombasa, Mombasa, Kenya; Department of Diagnostic Medicine/Pathobiology, College of Veterinary Medicine, Kansas State University, Manhattan, Kansas

## Abstract

In this cross-sectional seroepidemiological study we sought to examine the evidence for circulation of Rift Valley fever virus (RVFV) among herders in Madagascar and Kenya. From July 2010 to June 2012, we enrolled 459 herders and 98 controls (without ruminant exposures) and studied their sera (immunoglobulin G [IgG] and IgM through enzyme-linked immunosorbent assay [ELISA] and plaque reduction neutralization test [PRNT] assays) for evidence of previous RVFV infection. Overall, 59 (12.9%) of 459 herders and 7 (7.1%) of the 98 controls were positive by the IgG ELISA assay. Of the 59 ELISA-positive herders, 23 (38.9%) were confirmed by the PRNT assay (21 from eastern Kenya). Two of the 21 PRNT-positive study subjects also had elevated IgM antibodies against RVFV suggesting recent infection. Multivariate modeling in this study revealed that being seminomadic (odds ratio [OR] = 6.4, 95% confidence interval [CI] = 2.1–15.4) was most strongly associated with antibodies against RVFV. Although we cannot know when these infections occurred, it seems likely that some interepidemic RVFV infections are occurring among herders. As there are disincentives regarding reporting RVFV outbreaks in livestock or wildlife, it may be prudent to conduct periodic, limited, active seroepidemiological surveillance for RVFV infections in herders, especially in eastern Kenya.

## Introduction

Since its first discovery in 1931,^1^,^2^ Rift Valley fever virus (RVFV) has been detected in various sub-Saharan countries, as well as Egypt, Saudi Arabia, and Yemen, causing numerous outbreaks among both animals and humans.^3^–^6^ Kenya's most recent Rift Valley Fever (RVF) outbreak of 2006–2007 spread to multiple provinces and districts and resulted in nearly 400 cases of severe illness with at least 118 human deaths.^5^,^7^ Epidemiological data collected from some of the patients demonstrated that two-thirds were exposed to a recently ill animal before infection.^8^ In addition, data suggested that other risk factors, including drinking raw milk, owning an ill animal, working as a herdsman, and slaughtering an animal, were also associated with RVFV infection.^5^,^8^

From January to May 2008 and from November 2008 to March 2009, a RVFV strain, similar to that identified in the 2006–2007 outbreaks in Kenya, was identified as the causative agent in human and animal outbreaks across Madagascar, which resulted in a total of 26 human deaths.^9^ However, this was not the first epizootic to occur in Madagascar, as outbreaks were also reported in the east coast in 1990 and 1991, which resulted in increased abortion rates among pregnant cattle by 17% and 15%, respectively.^10^,^11^

Following these outbreaks, it has been strongly suggested that enhanced surveillance should be implemented to more effectively predict and respond to future outbreaks.^9^ Though positive gains have been made to monitor RVFV in these countries, little is known regarding the maintenance of the virus during interepidemic periods.^12^–^17^ In an effort to better understand the ecology of human RVFV infections, we conducted this cross-sectional, seroepidemiological study of persons with intense exposure to ruminants living in eastern Kenya, western Kenya, and Madagascar.

## Methods

### Study settings and design.

**Figure 1.**
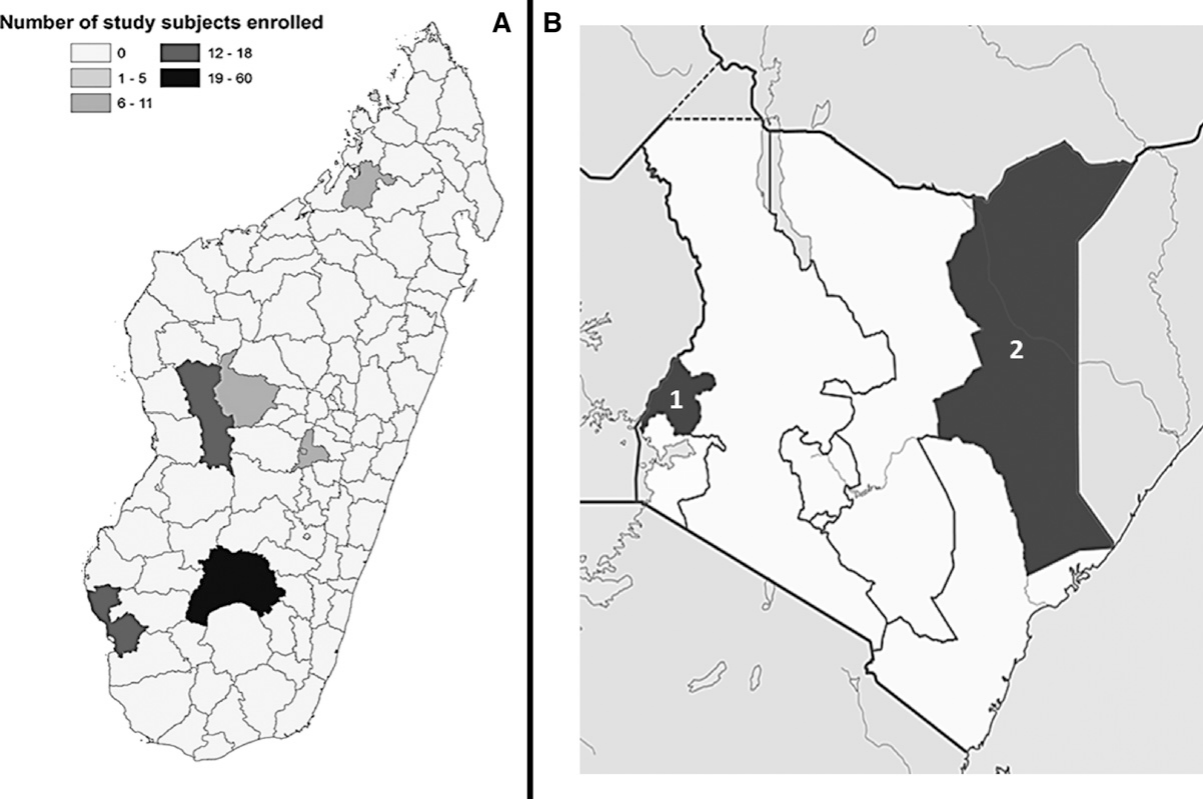
Maps of locations where study subjects were enrolled. (**A**) Location of study subjects in Madagascar. (**B**) Provinces in Kenya where study subjects were enrolled: 1, Western Province (*N* = 200 participants); 2, Garissa County (formerly North Eastern Province) (*N* = 230 participants).

This study was approved by Western Institutional Review Board and institutional review boards from collaborating institutions at each of the study sites (eastern Kenya—KEMRI Non-SSC no. 291, western Kenya—KEMRI SC1701, and Madagascar). Study personnel from each study site used informed consent to enroll participants ≥ 18 years of age who had a history of contact with ruminants. In Madagascar, participants were enrolled from the districts of Antsirabe, Antsohihy, Ihosy, Miandrivazo, Nosy Be, Toliara, Toliara II, and Tsiroanomandidy during the period January–March 2012 (Figure 1A

). In eastern Kenya, participants were enrolled from the villages of Gababa, Haji Mohamed, Hathama Chari, and Masalani in the North Eastern Province (Figure 1B) during February 6–12, 2012. In western Kenya, participants were enrolled from the Western Province (Figure 1B) during July 2010 to June 2012.

Ruminant exposure was defined as having an average of one or more cumulative hours per week exposure to camels, cattle, goats, or sheep, either by contact through touching and/or coming within 1 m of such animals during the 12 months before enrollment. Participants enrolled as controls resided in the same areas, denied having such contact, and when possible, were loosely age, group, and gender matched to exposed participants based on an expected final distribution of exposed study participants. Exclusion criteria for both groups included individuals less than 18 years of age, having any reported immunosuppression, or having been identified as medically likely to have greater susceptibility to various infectious agents.

### Sample collection.

Upon enrollment, participants completed an enrollment questionnaire with questions about demographics, animal and environmental exposures, and relevant medical information. Participants then permitted a serum sample collection, which was preserved at −80°C. Aliquots of serum were later shipped on dry ice to the University of Florida Emerging Pathogens Institute where they were first screened for human anti-RVF immunoglobulin G (IgG) antibodies with an enzyme-linked immunosorbent assay (ELISA). ELISA-positive samples were then tested with a plaque reduction neutralization test (PRNT) for validation. Finally, PRNT-positive samples were tested for human anti-RVF IgM antibodies with ELISA to delineate acute infections.

### IgG enzyme-linked immunosorbent assay.

Sera received from Madagascar and Kenya were first heat inactivated for 30 minutes at 56°C and then screened for IgG antibodies using a commercial RVFV human IgG ELISA kit obtained from Biological Diagnostic Supplies Limited (Scotland, United Kingdom) according to the manufacturer's instructions. In brief, plates were coated with a recombinant nucleocapsid RVFV antigen diluted 1:1,000 in sodium bicarbonate buffer (pH = 9.6), covered with plate seals, and incubated at 4°C overnight. Unbound antigen was removed by washing three times for 15 seconds each using phosphate-buffered saline with 0.05% Tween 20 (PBS-T). Plates were then blocked with 10% (w/v) skimmed milk powder (SM) in PBS at 37°C for 1 hour. Plates were washed with PBS-T, test sera added in duplicate at a dilution of 1:400 in PBS + 2% (w/v) SM, and incubated for 1 hour at 37°C. Plates were washed once more, and horseradish peroxidase (HRP)–conjugated antihuman IgG antibody, diluted 1:25,000 in PBS + 2% (w/v) SM, was added to each well and incubated for 1 hour at 37°C. After a final wash, chromogenic detection of HRP was performed by the addition of 0.1 mL of the peroxidase substrate 3,3′,5,5′-tetramethylbenzidine (TMB) (KPL, Inc., Gaithersburg, MD), at room temperature for 10 minutes and stopped by the addition of 0.1 mL 1 N sulfuric acid.^18^ Absorbance of each well at 450 nm (A_450_) was measured by a PowerWave HT microplate spectrophotometer (Biotek, Winooski, VT). Negative and positive control sera were included for each plate. Sera samples were considered positive if their optical density (OD) calculation was ≥ 0.29 (net OD serum/net mean OD positive control).

### Plaque reduction neutralization test.

All samples testing positive by the ELISA kit were further studied using PRNT adapted from methods previously described.^19^,^20^ RVFV MP-12 vaccine strain, propagated in Vero-CCL81 cells, was used in the PRNT assay. Sera were tested in duplicate using six 4-fold dilutions starting with 1:10 and ending at 1:10,240. A back titration of the diluted stock MP-12 virus was performed each time assays were run to ascertain the titer of virus stock used (typically, 30–60 plaque forming units/mL). A neutralization cutoff of 80% reduction, as determined by a corresponding back titration plate, was used to determine sera titration.

### IgM enzyme-linked immunosorbent assay.

To ascertain whether an individual had evidence of an acute infection, an indirect capture ELISA, adapted in-house following the principles of Paweska and others,^21^ was used. Because of having a limited amount of test serum and reagents, IgM testing was performed only for individuals who tested positive with the PRNT assay. First, 96-well microtiter plates were coated with a goat antihuman IgM antibody (catalog no. 01-10-03; KPL, Inc.) at a dilution of 1:2,000 in sodium bicarbonate buffer (pH = 9.6), covered with plate seals and incubated at 4°C overnight. Unbound antibody was washed from the well with PBS-T, and plates were then blocked with PBS with 5% (w/v) SM at room temperature for 2 hours. Test sera, diluted 1:100 in PBS-T plus 5% (w/v) SM, were added to coated plates and allowed to incubate for 1 hour at 37°C. Gamma-irradiated RVFV antigen, obtained from BEI Resources (National Institute of Allergy and Infectious Diseases, National Institutes of Health, RVFV, ZH501, Gamma-irradiated, NR-37380), was diluted 1:1,000 in PBS-T with 5% (w/v) SM, added to the plates, and allowed to incubate for 1 hour at 37°C. Rabbit anti-RVFV polyclonal antibody, obtained from Integrated Biotherapeutics Inc. (catalog no. 04-0001; Gaithersburg, MD), was diluted 1:1,000 in PBS-T with 5% SM, added to the plates, and allowed to incubate for 1 hour at 37°C. Extra serum adsorbed HRP-conjugated goat anti-rabbit IgG antibody (catalog no. 074-15-061, KPL, Inc.) was diluted 1:2,000, added to each well, and incubated for 1 hour at 37°C. All wells were washed five times after each incubation step using PBS-T. Each plate contained a “no antigen” negative control well to adjust for background absorbance. Chromogenic detection of HRP and plate reading was performed as described above. An IgM-positive control sample was not available for this assay. Instead, serum samples collected from six individuals with no possible RVFV exposure were collected and included in the assay run. IgM positivity was defined as any sample with an average A_450_ OD greater than three times the standard deviation plus the average A_450_ OD of the six negative control sera.

### Statistical analysis.

Bivariate χ^2^ tests of independence or Fisher's exact test were used to examine the association of demographic variables where PRNT serological outcomes were available. ELISA IgG positivity was used as the outcome variable when PRNT serological outcomes were not available. Variables determined by bivariate analyses to be statistically associated with RVFV seropositivity (*P* < 0.25) were then entered into a multivariable unconditional logistic regression model. Backward elimination was performed and covariates with *P* < 0.05 were retained in the model. Individual predictors retained in the final logistic models were tested for collinearity using bivariate χ^2^ tests. Finally, Hosmer–Lemeshow χ^2^ statistics for goodness of fit were performed. All demographic statistics, bivariate testing, and logistic modeling were conducted using SAS version 9.3 (SAS Institute, Cary, NC).

## Results

### Study population.

In Madagascar, participants were enrolled from the north, central, and south regions of the country, representing each of the unique climatic regions (Figure 1). Madagascar is an island nation with a population of nearly 23 million, located approximately 250 miles off the eastern coast of the African continent, south of the Equator. It is bordered to the west by the Mozambique Channel and to the east by the Indian Ocean. Participants were also enrolled from areas in the eastern and western regions of Kenya, a country of over 44 million in population. In eastern Kenya, individuals were enrolled in Garissa County, which is bordered by Somalia to the east and also where RVFV cases have been previously reported. The human populations in this area are seminomadic pastoralists, which depend on livestock herds for survival. In western Kenya, participants were enrolled in the formerly named Western Province, which is bordered by Lake Victoria to the south and Uganda to the west. This region of Kenya has a mixed crop–livestock farming system and a high human population density, with a heavy endemic and epidemic disease burden on both humans and animals. Western Kenya contains a range of ecological settings from the Lake Victoria system in the south to a semi-mountain system on the lower slopes of Mount Elgon in the north.

We enrolled 127 participants (93 exposed and 34 controls) from Madagascar, 230 participants (all exposed) from eastern Kenya, and 200 participants (136 exposed and 64 controls) from western Kenya (Table 1).

### ELISA and plaque reduction neutralization test.

**Figure 2.**
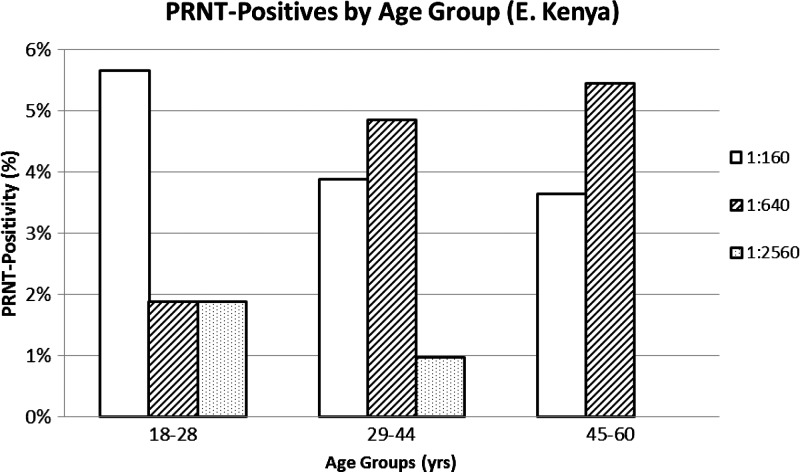
Distribution of plaque reduction neutralization test (PRNT) positives (%) by age group from eastern Kenya.

Of the 127 samples collected from Madagascar and tested by the ELISA assay, eight (6.3%) screened positive for IgG antibodies, of which two were confirmed positive by the PRNT assay at a sera dilution of 1:160 and 1:640. Between the two confirmed IgG PRNT-positive samples, both were from individuals with exposure to ruminants (two of 93 exposed = 2.15% RVFV positive) (Table 2). One of the two IgG PRNT-positive samples, which had a titer of 1:640, also tested positive by ELISA for IgM antibodies. This sample was collected from a man of 58 years, with daily reported exposure to cattle, who lived in Tsiroanomandidy and had no travel history outside Madagascar. This individual also reported monthly handling of raw meat and butchering, frequently sleeping outside close to his cattle, and reported regular exposure to mosquito bites. Despite being IgM positive, there were no reported symptoms of fever or being sick during the last 12 months. Of the 230 samples collected from eastern Kenya and tested by the ELISA assay, 36 (15.7%) screened positive for IgG antibodies. Of these 36 samples, 21 (58.3%) were confirmed positive by PRNT assay at a sera dilution ≥ 1:40. The titer range for the exposed confirmed positives was 1:160 to 1:2,560, and the age ranged from 18 to 65 years with a mean of 37.6 years (Table 2 and Figure 2

). Of the 200 samples collected from western Kenya and tested by the ELISA assay, 15 screened positive for IgG antibodies, though, none of these samples were confirmed positive by PRNT assay.

### Bivariate and multivariate analysis.

As a positive PRNT assay is less prone to cross-reactive biases, PRNT positivity was the outcome of choice for examining risk factor associations. Data from eastern Kenya were robust enough for examining PRNT as an outcome. However, as both Madagascar and western Kenyan sample populations yielded few PRNT positives, instead of PRNT, we examined ELISA IgG seropositivity as a surrogate for PRNT positivity. Bivariate and multivariate modeling results are recorded in Tables 3 and 4. Important bivariate risk factors for RVFV seropositivity included being seminomadic, drinking water from a public well or borehole, sleeping under a mosquito net, being bitten by a mosquito in the past 12 months, and wearing protective clothing when working with animals in the past 12 months (Table 3). Only the model for eastern Kenya yielded statistically significant risk factor associations with RVFV seropositivity: being seminomadic (odds ratio [OR] = 6.4, 95% confidence interval [CI] = 2.1–15.36) and sleeping under a mosquito net (OR = 3.2, 95% CI = 1.1–9.6). No collinearity problems were detected between any of the variables. Hosmer–Lemeshow χ^2^ statistics for goodness of fit indicated that predictors sufficiently described the data.

## Discussion

RVFV infections are considered as a major threat to the agricultural economies of many of the world's nations where competent mosquito vectors are endemic. Although previously contained in Africa and Middle East, experts have argued that considering modern transportation and trade routes, RVFV poses a large threat to the European Union and the United States.^22^,^23^ Realizing this threat, the U.S. government is investing considerable funding in understanding the ecology of RVFV and in developing better diagnostics and vaccines.^24^ As part of this effort, we conducted this cross-sectional seroepidemiological study to assess human evidence of RVFV infection.

In general, our seroepidemiological study did not yield evidence for many unrecognized human RVFV infections in the three geographical areas we examined. However, because some of our seropositive study subjects were relatively young, our data suggest that indeed some human RVFV infections may be occurring during interepidemic periods. This would support the notion that passive surveillance for RVFV is not highly effective in detecting new epidemic threats. Our findings are also remarkable in that seropositivity was confirmed only among those with exposures to ruminants, and the risk factors of being seminomadic are consistent with our understanding of the ecology of RVFV infections.

The use of bed netting to reduce exposure to mosquitoes also had a positive association with RVFV seropositivity, which may seem unexpected; however, it may be explained by confounders that were not assessed by our survey. Possible confounders include differences in vector species and behavior, particularly as it relates to host seeking and feeding. For example, the protective effect of bed netting would be markedly reduced if RVFV mosquito vectors in a given area were predominantly daytime biters or displayed exophagic behavior. In addition, it is possible that individuals who reported using bed netting were also more likely to be in areas with higher densities of mosquitoes, resulting in an overall increased risk of exposure. This finding underscores the need to better understand vector ecology as it applies to RVFV transmission in interepidemic areas, however, it is to be noted that bed nets remain an effective method for the prevention of vector-borne diseases of public health importance such as malaria.

Our study had several limitations. Its cross-sectional nature makes it difficult for us to understand when the RVFV infections may have occurred among study subjects. Also, while we can infer some exposure information by the age of the seropositive subject, the precision of this association is poor. Prospective studies are a much better approach to estimating risk of human infection over time. The study is also limited in that we used slightly different approaches in sampling (sample population from eastern Kenya were all exposed) and in questioning the study subjects (instruments differed slightly between sites).

Considering our three sampling areas in this work and another similar study we conducted in Saudi Arabia,^25^ our findings suggest that we are not missing large outbreaks of RVFV infections in the current surveillance and reporting systems. However, it does seem likely that small outbreaks, which affect both animals and man, may be missed or unreported. While one might argue that animals are the most sensitive sentinels for RVFV outbreaks, there are considerable disincentives for reporting RVFV outbreaks. Hence, conducting limited active surveillance for RVFV in man may be an important supplement to the surveillance that is conducted among domestic animals and wildlife. In particular, eastern Kenya would seem a good site to conduct such active surveillance for RVFV in herders. For instance, periodically screening subsets of herders for serological evidence of RVFV infection at various primary care clinics and hospitals in this region would seem a prudent and likely inexpensive additional early warning measure.

## Figures and Tables

**Table 1:** Demographic characteristics of the study participants enrolled in 2012, Madagascar and Kenya

Demographic characteristics	Madagascar		Eastern Kenya	Western Kenya	
	Exposed*	Nonexposed	Exposed*	Exposed*	Nonexposed
	*n* (%)	*n* (%)	*n* (%)	*n* (%)	*n* (%)
Total	93 (100)	34 (100)	230 (100)	136 (100)	64 (100)
Gender					
Male	82 (88)	11 (32)	76 (33)	61 (45)	25 (39)
Female	10 (11)	24 (71)	154 (67)	75 (55)	39 (61)
Age group (years)					
18–28	32 (34)	10 (29)	58 (25)	54 (40)	21 (33)
29–44	37 (40)	11 (32)	100 (44)	39 (29)	22 (34)
45–60	18 (19)	10 (29)	55 (24)	25 (18)	9 (14)
> 60	5 (5)	4 (12)	17 (7)	18 (13)	12 (19)
Madagascar (districts)					
Antsirabe	8 (9)	2 (6)	–	–	–
Antsohihy	5 (5)	3 (9)	–	–	–
Ihosy	44 (47)	16 (47)	–	–	–
Miandrivazo	10 (11)	9 (26)	–	–	–
Nosy Be	4 (4)	1 (3)	–	–	–
Toliara	0 (0)	1 (3)	–	–	–
Toliara II	12 (13)	1 (3)	–	–	–
Tsiroanomandidy	9 (10)	2 (6)	–	–	–
Eastern Kenya (village)					
Gababa	–	–	34 (15)	–	–
Haji Mohamed	–	–	150 (65)	–	–
Hathama Chari	–	–	17 (7)	–	–
Masalani	–	–	29 (13)	–	–
Western Kenya (tribe)					
Japadhola	–	–	–	0 (0)	1 (1.5)
Kikuyu	–	–	–	0 (0)	1 (1.5)
Luhya	–	–	–	63 (9)	22 (34)
Luo	–	–	–	29 (5)	17 (27)
Samia	–	–	–	21 (48)	6 (9)
Teso	–	–	–	23 (11)	17 (27)

* Exposure was defined as close contact through touching and/or coming within 1 m of a ruminant animal during the 12 months before enrollment.

**Table 2:** Demographic and exposure characteristics of the 23 study subjects PRNT-positive for antibodies against RVFV

No.	Site enrolled	Ruminant exposed*	Gender	Age group	PRNT titer	IgM positive
1	Madagascar	Yes	Male	45–60	1:640	Yes
2	Madagascar	No	Male	45–60	1:160	No
3	Eastern Kenya	Yes	Male	45–60	1:640	No
4	Eastern Kenya	Yes	Female	29–44	1:160	No
5	Eastern Kenya	Yes	Female	29–44	1:640	No
6	Eastern Kenya	Yes	Female	45–60	1:640	No
7	Eastern Kenya	Yes	Female	29–44	1:160	No
8	Eastern Kenya	Yes	Female	29–44	1:640	No
9	Eastern Kenya	Yes	Female	29–44	1:160	No
10	Eastern Kenya	Yes	Male	29–44	1:160	No
11	Eastern Kenya	Yes	Female	29–44	1:640	No
12	Eastern Kenya	Yes	Female	> 60	1:160	No
13	Eastern Kenya	Yes	Female	29–44	1:2,560	No
14	Eastern Kenya	Yes	Female	45–60	1:640	No
15	Eastern Kenya	Yes	Female	45–60	1:160	No
16	Eastern Kenya	Yes	Male	18–28	1:640	No
17	Eastern Kenya	Yes	Female	18–28	1:2,560	No
18	Eastern Kenya	Yes	Male	18–28	1:160	No
19	Eastern Kenya	Yes	Female	18–28	1:160	No
20	Eastern Kenya	Yes	Female	29–44	1:640	No
21	Eastern Kenya	Yes	Female	18–28	1:160	No
22	Eastern Kenya	Yes	Male	29–44	1:640	No
23	Eastern Kenya	Yes	Male	45–60	1:160	No

IgM = immunoglobulin M; PRNT = plaque reduction neutralization test; RVFV = Rift Valley fever virus.

* Exposure was defined as close contact through touching and/or coming within 1 m of a ruminant animal during the 12 months before enrollment.

**Table 3:** Unadjusted ORs for risk factors associated with evidence of previous RVFV infection based on ELISA IgG seropositivity (Madagascar and western Kenya)

Risk factor	Madagascar				Western Kenya			
	Total *N*	No. (%)	Unadjusted OR (95% CI)	Adjusted OR (95% CI)	Total *N*	No. (%)	Unadjusted OR (95% CI)	Adjusted OR (95% CI)
Ruminant exposure*								
Yes	93	6 (6.5)	1.1 (0.21, 5.8)	–	136	10 (7.4)	0.94 (0.31, 2.9)	–
No	34	2 (5.9)	Ref.		64	5 (7.8)	Ref.	
Age (years)								
18–28	42	3 (7.1)	1.2 (0.28, 5.4)	–	75	6 (8.0)	1.1 (0.38, 3.3)	–
29–44	48	0 (0)	–		61	3 (4.9)	0.55 (0.15, 2.0)	
45–60	28	4 (14.3)	4.0 (0.92, 17.0)		34	2 (5.9)	0.74 (0.16, 3.4)	
> 60	9	1 (11.1)	2.0 (0.22, 18.2)		30	4 (13.3)	2.2 (0.66, 7.5)	
Gender								
Female	34	2 (5.9)	0.91 (0.17, 4.7)	–	114	7 (6.1)	0.64 (0.22, 1.8)	–
Male	93	6 (6.5)	Ref.		86	8 (9.3)	Ref.	
Seminomadic								
Yes	1	0 (0)	–	–	N/A	N/A	N/A	N/A
No	125	8 (6.4)						
Drinking water from public well/borehole								
Yes	58	3 (5.2)	0.70 (0.16, 3.05)	–	83	6 (7.2)	0.94 (0.32, 2.7)	–
No	69	5 (7.2)	Ref.		117	9 (7.7)	Ref.	
Sleep under a mosquito net								
Yes	96	5 (5.2)	0.51 (0.12, 2.28)	–	N/A	N/A	N/A	N/A
No	31	3 (9.7)	Ref.					
Bitten by a mosquito in past 12 months								
Yes	123	7 (5.7)	0.51 (0.12, 2.3)	–	N/A	N/A	N/A	N/A
No	4	1 (25.0)	Ref.					
Wear protective clothing when working with animals in past 12 months								
Yes	3	0 (0)	–	–	N/A	N/A	N/A	N/A
No	116	8 (6.9)						
Cared for birthing animal in past 12 months								
Yes	2	0 (0)	–	–	19	0 (0)	–	–
No	124	8 (6.9)			181	15 (8.3)		
Butchered animal in past 12 months								
Yes	67	5 (7.5)	1.5 (0.35, 6.7)	–	34	3 (8.8)	1.2 (0.33, 4.7)	–
No	60	3 (5.0)	Ref.		166	12 (7.2)	Ref.	
Reported fever in past 12 months								
Yes	46	4 (8.7)	1.8 (0.44, 7.7)	–	120	8 (6.7)	0.74 (0.26, 2.1)	–
No	81	4 (4.9)	Ref.		80	7 (8.8)	Ref.	

CI = confidence interval; ELISA = enzyme-linked immunosorbent assay; IgG = immunoglobulin G; N/A = data were not available for this covariate; OR = odds ratio; RVFV = Rift Valley fever virus; Ref. = Reference.

* Exposure was defined as close contact through touching and/or coming within 1 m of a ruminant animal during the 12 months before enrollment.

**Table 4:** Unadjusted and adjusted ORs for risk factors associated with evidence of previous RVFV infection by elevated PRNT assay (eastern Kenya)

Risk factor	Eastern Kenya			
	Total *N*	No. (%)	Unadjusted OR (95% CI)	Adjusted OR (95% CI)
Ruminant exposure*				
Yes	0	0 (0)	–	–
No	230	21 (9.1)		
Age (years)				
18–28	53	4 (7.5)	0.95 (0.40, 2.2)	–
29–44	103	10 (9.7)	0.75 (0.36, 1.6)	
45–60	55	5 (9.1)	1.8 (0.81, 3.8) 0.32 (0.04, 2.5)	
> 60	17	1 (5.9)	–	–
Gender				
Female	154	15 (9.7)	1.3 (0.5, 3.4)	–
Male	76	6 (7.9)	Ref.	
Seminomadic				
Yes	77	15 (19.5)	5.9 (2.2, 16.0)	6.4 (2.1, 15.4)
No	153	6 (3.9)	Ref.	Ref.
Drinking water from public well/borehole				
Yes	65	11 (16.9)	3.2 (1.3, 7.9)	–
No	165	10 (6.0)	Ref.	
Sleep under a mosquito net				
Yes	27	6 (22.2)	3.6 (1.3, 10.2)	3.2 (1.1, 9.6)
No	203	15 (7.4)	Ref.	Ref.
Bitten by a mosquito in past 12 months				
Yes	116	15 (12.9)	2.7 (1.0, 7.2)	–
No	114	6 (5.3)	Ref.	
Wear protective clothing when working with animals in past 12 months				
Yes	17	4 (23.5)	3.5 (1.0, 11.9)	–
No	210	17 (8.1)	Ref.	
Cared for birthing animal in past 12 months				
Yes	224	20 (8.9)	0.39 (0.04, 3.7)	–
No	5	1 (20.0)	Ref.	
Butchered animal in past 12 months				
Yes	212	20 (9.4)	1.6 (0.2, 12.5)	–
No	16	1 (6.3)	Ref.	
Reported fever in past 12 months				
Yes	186	14 (7.5)	0.43 (0.16, 1.1)	–
No	44	7 (15.9)	Ref.	

CI = confidence interval; PRNT = plaque reduction neutralization test; OR = odds ratio; RVFV = Rift Valley fever virus.

* Exposure was defined as close contact through touching and/or coming within 1 m of a ruminant animal during the 12 months before enrollment.
